# Stability-Indicating HPLC Method for the Determination of Cefcapene Pivoxil

**DOI:** 10.1007/s10337-012-2318-1

**Published:** 2012-09-18

**Authors:** Przemysław Zalewski, Judyta Cielecka-Piontek, Piotr Garbacki, Anna Jelińska, Marta Karaźniewicz-Łada

**Affiliations:** 1 Department of Pharmaceutical Chemistry, Faculty of Pharmacy, Poznan University of Medical Sciences, Grunwaldzka 6, 60-780 Poznań, Poland; 2 Department of Physical Pharmacy and Pharmacokinetics, Faculty of Pharmacy, Poznan University of Medical Sciences, Grunwaldzka 6, 60-780 Poznań, Poland

**Keywords:** Column liquid chromatography, Stability-indicating method, Cefcapene pivoxil

## Abstract

The stability-indicating LC assay method was developed and validated for quantitative determination of cefcapene pivoxil in the presence of degradation products formed during forced degradation studies. An isocratic RP-HPLC method was developed with a Lichrospher RP-18 (250 mm × 4.6 mm, 5 μm) column and the mobile phase composed of 45 volumes of acetonitrile and 55 volumes of mixture composed of citric acid 10 mmol L^−1^ and potassium chloride 18 mmol L^−1^. The flow rate of the mobile phase was 1 mL min^−1^. Detection wavelength was 270 nm and temperature was 30 °C. Cefcapene pivoxil, similar to other cephalosporins, was subjected to stress conditions of degradation in aqueous solutions including hydrolysis, oxidation, and thermal degradation. The method was validated with regard to linearity, accuracy, precision, selectivity, and robustness. The method was applied successfully for the determination of cefcapene pivoxil during kinetic studies in aqueous solutions (pH and thermal degradation) and in solid state (oxidative, thermal, and radiolytic degradation).

## Introduction

Cefcapene pivoxil is a new, oral, third-generation cephalosporin. It has a broad spectrum of antibacterial activity against Gram-positive and Gram-negative bacteria, including *Staphylococcus aureus* [1]. Cefcapene pivoxil contains a carbamoyloxymethyl group at the C3 position, determining its antibacterial activity against *Staphylococcus aureus*. It was originally created by Shionogi & Co., Ltd. and launched as Flomox in 1997. Previous studies proved that cephalosporins are susceptible to degradation in aqueous solutions [2–4] and in the solid state [5–9].

An essential parameter affecting the safety of drug use is chemical stability. The antibacterial activity of cephalosporins is attributed to their β-lactam moieties, which are very susceptible to chemical degradation. Most side effects of β-lactams are caused by their degradation products that hinder the development of analytical methods for the determination of cephalosporins. An optimal method is expected to separate and determine the substance to be examined in the presence of related products such as in-process impurities, degradation products, and metabolites [10, 11]. Although various methods are known for the determination of cefcapene pivoxil in biological fluids (blood, tissue, and cerebrospinal fluid) [12, 13], they are selective only for the metabolites of cefcapene pivoxyl. The current guidelines of the International Conference on Harmonization (ICH) require the development of stability-indicating assay methods (SIAMs) suitable for the determination of drugs based on the analysis of stability test samples (Q1A–R2) (ICH Q2B, validation of analytical procedures, methodology). The stress tests are required to establish the effect of temperature and humidity in the solid state, the impact of temperature, light, oxidizing agent, pH, buffer, and infusion fluids in solutions and the influence of biochemical processes on the formation of metabolites [14].

Microbiological methods have traditionally been used for determining cephalosporin. Yet, they are time-consuming and unable to differentiate antibiotics. HPLC methods, as opposed to microbiological techniques are rapid and specific. The aim of this work was to develop and validate an HPLC method with UV detection suitable for the identification, determination, and stability studies of cefcapene pivoxil.

## Experimental

### Standards and Reagents

Cefcapene pivoxil hydrochloride was obtained from Bepharm Ltd., 128 Xiangyin Road, Yangpu District, Shanghai, China. It is white or pale yellowish white crystalline powder slightly soluble in water.

All other chemicals and solvents were obtained from Merck KGaA (Germany) and were of analytical grade. High quality pure water was prepared using the Millipore purification system (Millipore, Molsheim, France, model Exil SA 67120).

### Instrumentation

The analytical system consisted of a quaternary pump (L-7100), an autosampler (L-7200), a column oven (L-7360), and a diode array detector (L-7455) (all Merck Hitachi products). As the stationary phase, a Lichrospher RP-18 column, 5 μm particle size, 250 mm × 4 mm (Merck, Darmstadt, Germany) was used. The mobile phase composed of 45 volumes of acetonitrile and 55 volumes of mixture composed of citric acid 10 mmol L^−1^ and potassium chloride 18 mmol L^−1^, pH of the mobile phase was 2.36. The flow rate of the mobile phase was 1 mL min^−1^. The wavelength of the DAD detector was set at 270 nm. Separation was performed at 30 °C.

## Procedure for Forced Degradation Study of Cefcapene Pivoxil

### Degradation in Aqueous Solutions

The degradation of cefcapene pivoxil in aqueous solutions was studied at 363 K in hydrochloric acid (0.3 mol L^−1^). The ionic strength of all solutions was adjusted to 0.5 mol L^−1^ with a solution of sodium chloride (4 mol L^−1^). Degradation was initiated by dissolving an accurately weighed 10 mg of cefcapene pivoxil in 50 mL of the solution equilibrated to 363 K in stopped flasks. At specified times, samples of the reaction solutions (1 mL) were instantly cooled with a mixture of ice and water.

### Oxidative Degradation

Degradation was initiated by dissolving an accurately weighed 10 mg of cefcapene pivoxil in 50 mL of 30 % H_2_O_2_ solution equilibrated to 343 K in stopped flasks. At specified times, samples of the reaction solutions (1 mL) were instantly cooled with a mixture of ice and water.

### Thermal Degradation

5 mg of samples of cefcapene pivoxil was weighed in 5 mL vials. In order to achieve the degradation of cefcapene pivoxil in solid state, their samples were immersed in heat chambers at 373 or 393 K, both at RH = 0 %. At specified time intervals, determined by the rate of degradation, the vials were removed, cooled to room temperature and their contents were dissolved in acetonitrile. The obtained solutions were quantitatively transferred into measuring flasks and diluted with acetonitrile to 25 mL.

### Radiolytic Degradation

5 mg of samples of cefcapene pivoxil was weighed in 5 mL vials and closed with a plastic stopper. The samples in the vials were exposed to irradiation in a linear electron accelerator LAE 13/9 (electron beam 9.96 MeV and current intensity 6.2 μA) till they absorbed a dose of 25 and 400 kGy. The vials were removed and their contents were dissolved in acetonitrile. The obtained solutions were quantitatively transferred into measuring flasks and diluted with acetonitrile to 25 mL.

## Results and Discussion

### Optimization of Chromatographic Conditions

**Fig. 1:**
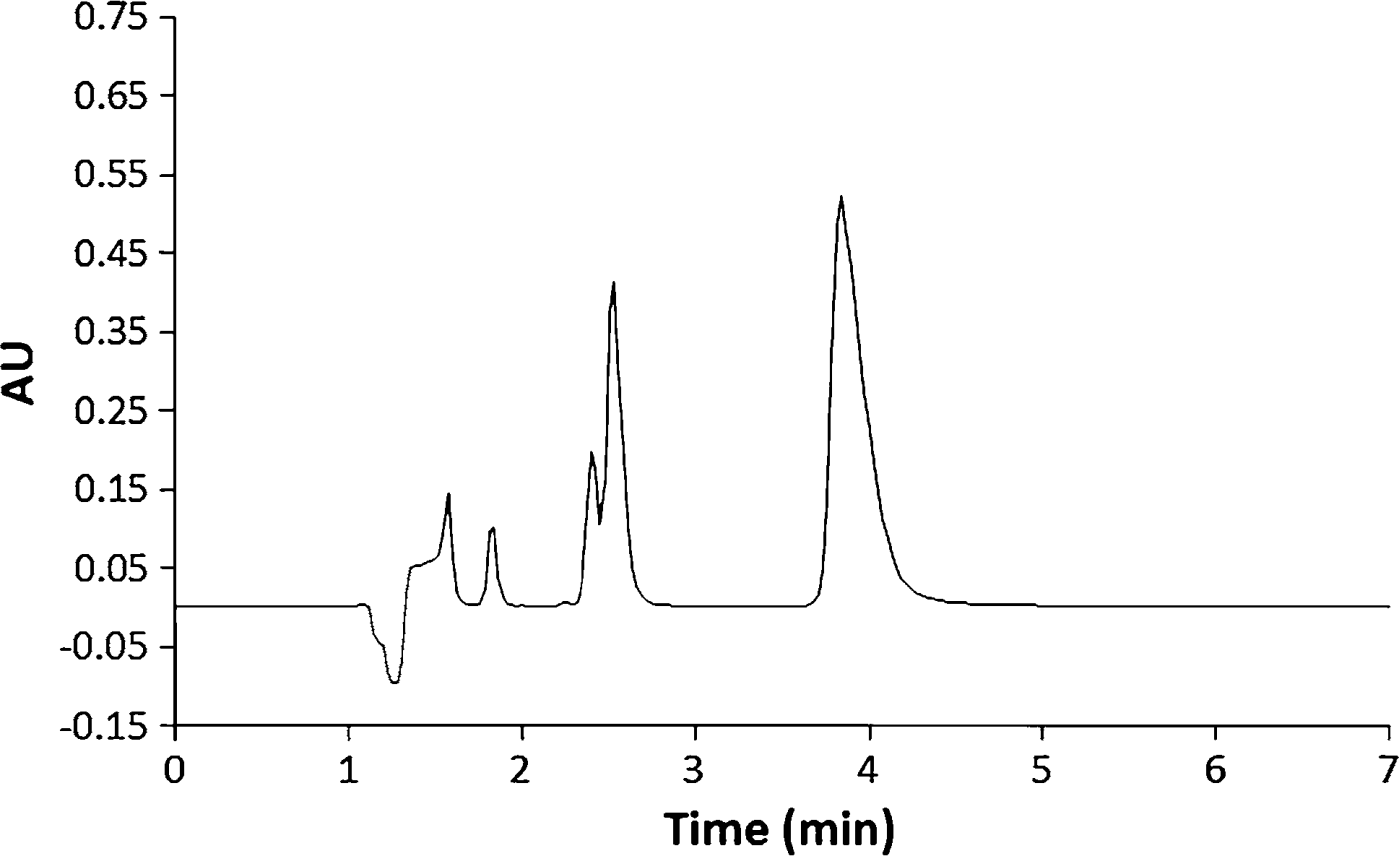
The HPLC chromatogram of cefcapene pivoxil (*t*_R_ = 3.84 min) in the presence of degradation products (*t*_R_ from 1.57 to 2.53 min) following incubation at 0.5 mol L^−1^ HCl, at 363 K for 180 min

In HPLC method with mobile phase composed of 70 volumes of mixture composed of potassium dihydrogen phosphate 1.56 g L^−1^, sodium dodecyl sulfate 1.22 g L^−1^, 30 volumes of acetonitrile and 10 volumes of methanol separation was unsatisfactory. In HPLC method with mobile phase composed of 40 volumes of acetonitrile and 60 volumes of mixture composed of citric acid 50 mmol L^−1^ and potassium chloride 30 mmol L^−1^ adjusted to pH 3 by sodium hydroxide [12] peak asymmetry and resolutions were unsatisfactory. Change to pH 2.4 (without additions of sodium hydroxide) and in concentration of citric acid, potassium chloride and acetonitrile were satisfactory changing resolutions and peak asymmetry. It was observed that satisfactory resolution of cefcapene pivoxil (retention time 3.84 min) and their degradation products (retention time from 1.57 to 2.53 min) formed under various stress conditions was achieved when analysis of stressed samples was performed on an HPLC system using a C-18 column and a mobile phase composed of 45 volumes of acetonitrile and 55 volumes of mixture composed of citric acid 10 mmol L^−1^ and potassium chloride 18 mmol L^−1^. The detection was carried out at 270 nm. The mobile phase flow rate was 1 mL min^−1^. Typical retention times of cefcapene pivoxil were about 3.84 min (Fig. 1). In blank sample, purity of peak of cefcapene pivoxil was 100 % and peak asymmetry was 1.95.

## Method Validation

HPLC method was validated according to the International Conference on Harmonization Guidelines (ICH Q2B, validation of analytical procedures, methodology). The method was validated for parameters such as selectivity, linearity, precision, accuracy, and robustness.

### Selectivity

The selectivity was examined for non-degraded and degraded samples [the solutions of cefcapene pivoxil after stress conditions of acid hydrolysis at 363 K, oxidation (H_2_O_2_), and thermal degradation (373 and 393 K)].

The HPLC method for determination of cefcapene pivoxil was found selective in the presence of degradation products as shown in Fig. 1. Peaks were symmetrical, clearly separated from each other (Fig. 1). Photodiode array detection was used as an evidence of the specificity of the method and to evaluate the homogeneity of cefcapene pivoxil peaks. The peak purity values were more than 98.79 % for cefcapene pivoxil at 270 nm, what proves that degradants were not interfering with the mean peak (Table 1).

**Table 1:** Results of forced degradation studies

Stress conditions and time studies	Degradation (%)	Peak purity^a^
Acidic/0.5 mol L^−1^ HCl/363 K/240 min	56.4	100.00
Oxidizing/30 % H_2_O_2_/343 K/310 min	88.7	98.79
Thermal/373 K/28 days	9.4	100.00
Thermal/393 K/28 days	30.9	100.00
Radiolytic/25 kGy	1.7	99.98
Radiolytic/400 kGy	10.8	99.15

^a^Peak purity values in the range of 98.5–100 indicates a homogeneous peak

### Linearity

Linearity was evaluated in the concentration range 20–240 mg L^−1^ (10–120 % of the nominal concentration of cefcapene pivoxil during degradation studies). The samples of each solution were injected three times and each series comprised six experimental points.

The calibration plots were linear in the following concentration range 20–240 mg L^−1^ (*n* = 6, *r* = 0.9992). The calibration curve was described by the equation *y* = *ac*; *y* = (5491604 ± 239226)*c*. The *b* value, calculated from equation *y* = a*c* + *b,* was insignificant because it was lower than the critical value *t*_b_ = *b*/*S*_b_. Statistical analysis using Mandel’s fitting test confirmed linearity of the calibration curves.

### Precision

Precision of the assay was determined in relation to repeatability (intra-day) and intermediate precision (inter-day). In order to evaluate the repeatability of the methods, six samples were determined during the same day for three concentrations of cefcapene pivoxil. Intermediate precision was studied by comparing the assays performed on two different days.

The intra-day and inter-day precision values of measured concentration of cefcapene pivoxil, as calculated from linearity plots are given in Table 2. The RSD values were 0.58 and 1.27 %, respectively, demonstrating that the method was precise.

**Table 2:** Intra-day, inter-day precision (*n* = 6) and recovery (*n* = 3) studies

Spiked concentration (mg L^−1^)	Measured concentration ± SD (mg L^−1^)	RSD (%)
Intra-day precision		
160.0	160.46 ± 0.78	0.50
200.0	200.21 ± 1.13	0.58
240.0	240.79 ± 1.09	0.47
Inter-day precision		
200.0	201.22 ± 2.47	1.27
Recovery studies		
160.0 (~80 %)	160.15 ± 0.65	100.09
200.0 (~100 %)	200.25 ± 1.03	100.12
240.0 (~120 %)	240.69 ± 1.41	100.29

Good recoveries were obtained for each concentration, confirming that the method was accurate (Table 2).

### Accuracy as Recovery Test

The accuracy of the method was determined by recovering cefcapene pivoxil from the placebo. The recovery test was performed at three levels 80, 100, and 120 % of the nominal concentration of cefcapene pivoxil during degradation studies. Three samples were prepared for each recovery level. The solutions were analyzed and the percentage of recoveries was calculated (Table 2).

### Limits of Detection (LOD) and Quantification (LOQ)

The LOD and LOQ parameters were determined from the regression equation of cefcapene pivoxil: LOD = 3.3 *S*_*y*_/*a*, LOQ = 10 *S*_*y*_/*a,* where *S*_*y*_ is a standard error and *a* is the slope of the corresponding calibration curve.

Under applied chromatographic conditions, the LOD of cefcapene pivoxil was 4.24 mg L^−1^ and LOQ of cefcapene pivoxil was 12.85 mg L^−1^.

### Robustness

The robustness of the procedure was evaluated after changing the following parameters: the composition of the mobile phase (concentration of acetonitrile in the range 43–47 %, concentration of citric acid in the range 9–11 mmol L^−1^, concentration of potassium chloride in the range 16–20 mmol L^−1^), the pH of mobile phase in the range 2.34–2.38, the mobile phase flow rate (flow rate in the range 1.48–1.52 mL min^−1^), wavelength of absorption (270 ± 5 nm), temperature (30 ± 2 °C). For each parameter change its influence on the retention time (*t*_R_), resolution (*R*_S_), area (*A*) and asymmetry of peak was evaluated (Table 3). No significant changes in resolution, retention time, area and asymmetry of peak were observed and tested parameters were modified.

**Table 3:** Results of robustness studies

Parameter	*t*_R_	*R*_S_^a^	*A*	Peak asymmetry^b^
Optimal	4.27	1.87	3817826	1.95
ACN = 47 %	4.32	1.75	3807469	1.85
ACN = 43 %	4.32	1.92	3813561	1.98
Citric acid = 11 mmol L^−1^	3.95	1.64	3881498	1.87
Citric acid = 9 mmol L^−1^	3.95	1.53	3872026	1.69
Potassium chloride = 20 mmol L^−1^	4.19	1.85	3798476	1.92
Potassium chloride = 16 mmol L^−1^	4.16	1.75	3812361	1.88
pH 2.38	4.32	1.80	3700756	1.90
pH 2.34	4.32	1.83	3850258	1.92
*f* = 1.52 mL min^−1^	4.27	1.79	3766090	1.91
*f* = 1.48 mL min^−1^	4.37	1.75	3835079	1.89
*λ* = 275 nm	4.32	1.86	3835709	1.94
*λ* = 265 nm	4.32	1.86	3827682	1.95
*T* = 28 °C	4.32	1.87	3811839	1.95
*T* = 32 °C	4.32	1.87	3810980	1.95

^a^Peaks are separated to baseline if resolution is >1.5

^b^Peak asymmetry <1.50 indicatives symmetry of peak

## Results of Forced Degradation Experiments

During stability studies, a 20–80 % degradation of the substance to be examined is to be achieved to qualify the assay method as able to indicate stability [14]. In previous studies, concerning the stability of cephalosporin reactions of acidic and basic hydrolysis occurred rapidly [4–8]. Cefcapene pivoxil was observed to be significantly degraded as a result of acidic hydrolysis and oxidative stress conditions at an increased temperature, although it was found to be relatively resistant to acidic hydrolysis at room temperature. After 240 min, 56.4 % of cefcapene pivoxil degraded in 0.5 mol L^−1^ HCl, at 363 K. Chromatograms of solutions obtained after degradation under acidic conditions are shown in Fig. 1. The main degradation products had retention times of about 1.57 and 2.53 min. Since cefcapene pivoxil precipitated in alkaline solutions and it was impossible to estimate its degradation rate during basic hydrolysis. Cefcapene pivoxil proved to be fairly resistant to oxidative stress conditions. To accelerate degradation, an increased temperature was used. Cefcapene pivoxil showed desired stability in dry hot air (373 and 393 K after 28 days). The main degradation products had the same retention time as those observed during acidic hydrolysis and oxidative degradation. Cefcapene pivoxil was also resistant to radiolytic stress conditions where 1.66–10.84 % of it is degraded. The results of forced degradation experiments on cefcapene pivoxil under various stress conditions are summarized in Table 1.

## Conclusions

The isocratic RP-LC method developed for the analysis of cefcapene pivoxil in their pharmaceutical preparations is selective, precise, and accurate. The method is useful for routine analysis due to short run time. This method can be used for determining the stability of cefcapene pivoxil in its pharmaceutical preparations.
